# Machine Learning in Predicting and Optimizing Polymer Printability for 3D Bioprinting

**DOI:** 10.3390/polym17131873

**Published:** 2025-07-04

**Authors:** Junjie Yu, Danyu Yao, Ling Wang, Mingen Xu

**Affiliations:** 1 School of Automation, Hangzhou Dianzi University, Hangzhou 310018, China; 242060294@hdu.edu.cn (J.Y.);; 2 Provincial Key Laboratory of Medical Additive Manufacturing and Information Integration, Hangzhou Dianzi University, Hangzhou 310018, China

**Keywords:** 3D bioprinting, printability, machine learning, polymer materials

## Abstract

Three-dimensional (3D) bioprinting has emerged as a highly promising technology within the realms of tissue engineering and regenerative medicine. The assessment of printability is essential for ensuring the quality of bio-printed constructs and the functionality of the resultant tissues. Polymer materials, extensively utilized as bioink materials in extrusion-based bioprinting, have garnered significant attention from researchers due to the critical need for evaluating and optimizing their printability. Machine learning, a powerful data-driven technology, has attracted increasing attention in the evaluation and optimization of 3D bioprinting printability in recent years. This review provides an overview of the application of machine learning in the printability research of polymers for 3D bioprinting, encompassing the analysis of factors influencing printability (such as material and printing parameters), the development of predictive models, and the formulation of optimization strategies. Additionally, the review briefly explores the utilization of machine learning in predicting cell viability, evaluates the advanced nature and developmental potential of machine learning in 3D bioprinting, and examines the current challenges and future trends.

## 1. Introduction

As an additive manufacturing technique, three-dimensional (3D) bioprinting is designed to fabricate organs or tissues through layer-by-layer deposition of bioinks, creating 3D functional tissue constructs. By leveraging controllable and reproducible technologies, 3D bioprinting generates the complex microstructures, offering innovative solutions for tissue engineering and regenerative medicine [1,2]. Currently, the predominant printing methods in 3D bioprinting include droplet-based, extrusion-based, and laser-based bioprinting technologies, with extrusion-based bioprinting being the most extensively utilized in tissue engineering and regenerative medicine [3,4,5,6,7].

Bioink is the cornerstone for constructing tissues and organs, and occupies a core position in the field of 3D bioprinting. Typically, bioink consists of a combination of various biological materials and must exhibit superior printability to accurately form and maintain the predefined 3D structure during the bioprinting process, thereby supporting cell viability and proliferation [8,9]. The components of common bioink materials are shown in Table 1. Among them, polymers are widely used in extrusion-based bioprinting due to their controllable physical and chemical properties and good biocompatibility.

Depending on the type of materials being printed, different extrusion methods should be applied. According to the driving mechanism, extrusion-based bioprinting can be divided into three types: pneumatic-, piston-, and screw-based bioprinting [28]. Pneumatic-based printing extrudes materials through compressed air and is suitable for viscoelastic bioinks with a wide rheological range, with the printing performance depending on the flowability and shear recovery ability of the ink [29]. In contrast, piston-based printing and screw-based printing adopt mechanical driving methods, which can handle bioinks with higher viscosity and achieve a more precise printing process by directly controlling the extrusion volume, with key parameters being the yield stress and storage modulus of the ink [30]. For example, synthetic polymers such as polyvinyl alcohol (PVA) and polycaprolactone (PCL), which possess high viscosity and thermal processing properties, are usually extruded using a melt deposition modeling-based method, where stable deposition and shaping are realized through heating and melting [31,32]. Slurries (such as ceramic slurries), due to their high solid content and shear-thinning properties, are suitable for extrusion driven by screw or piston systems [33]. For hydrogels, apart from shear thinning, their stability under shear and reversible destruction are also important, and generally employ pneumatic or mechanical piston-driven extrusion systems [34,35,36]. This review focuses on the evaluation and optimization of the printability of bioinks composed of polymer materials (including natural polymers and synthetic polymers).

The printability of bioink is a crucial issue in the field of 3D printing, directly influencing the quality, precision, and functionality of the printed structure. Printability serves as a key indicator for assessing the suitability of bioink for bioprinting applications, often evaluated by comparing the actual printed structure with the theoretical design [37,38]. Poor printability can lead to deformation or collapse of the printed structure, resulting in deviations from the desired shape and precision, thereby compromising the intended functionality. Therefore, accurate evaluation and optimization of printability are essential for constructing high-quality tissue engineering scaffolds.

However, in current research, researchers have devised diverse evaluation methods based on their specific research objectives. Although these methods are effective in specific scenarios, the absence of unified standards may compromise the comparability and generalizability of research findings. Moreover, the rapid advancements in printing technologies and biological materials have rendered traditional experimental methods for studying printability increasingly inadequate to meet research needs. Characterized by time-consuming procedures and low efficiency, traditional approaches struggle to keep pace with the evolving demands of contemporary research. Consequently, there is a pressing need for alternative approaches that can enhance research efficiency.

To address this challenge, researchers are exploring new strategies, such as integrating computational simulations, rheological analysis, and high-throughput experimentation, to predict and optimize bioink behavior during the printing process. The rise of machine learning has provided researchers with a new approach. By leveraging data-driven technologies, machine learning can replace traditional methods for addressing common printing issues, such as printability and cell viability [39,40,41]. Through the analysis of large and complex datasets, machine-learning algorithms can uncover underlying patterns and provide robust support for printability evaluation and prediction of printed structure performance, thereby accelerating bioprinting research [42,43]. For example, Mohammad Shirmohammadi et al. [44] used a hybrid artificial neural network algorithm to optimize the input parameters for achieving optimal surface roughness. Mohd Sazli Saad et al. [45] fitted the printing speed, layer height, and printing temperature using a regression model to obtain the minimum roughness. These applications demonstrate the great potential and superiority of machine learning in enhancing optimization efficiency and experimental speed.

This review provides a comprehensive summary of recent research on the printability of polymers in 3D bioprinting using machine learning. It highlights the potential of machine learning in tissue engineering, emphasizing the benefits of data-driven approaches in printability research. Additionally, it offers constructive recommendations for future studies on printability to accelerate the rapid advancement of 3D bioprinting technology.

## 2. Printability

### 2.1. Evaluation of Printability

The evaluation of printability is a crucial step in bioprinting, and its results have a significant impact on the functional performance of the printed constructs. Optimal printability is essential not only for achieving geometric precision and desirable mechanical properties but also for supporting subsequent biological functions, such as cell viability and tissue formation. However, due to the diverse experimental designs and research objectives among studies, the evaluation of printability currently relies on context-specific processes, and a unified standard has yet to be established [46]. Table 2 summarizes several common evaluation methods employed in current research.

As shown in Table 2, current evaluation methods primarily focus on two aspects: the accuracy of the printed filament width and the accuracy of the printed structure. These methods are generally applicable to most scaffold printing scenarios. With the continuous advancement of printing technologies, an increasing number of novel evaluation schemes are emerging to address the research needs associated with the design of increasingly complex printed structures and the performance assessment of scaffolds.

### 2.2. Factors Affecting Printability

**Figure 1:**
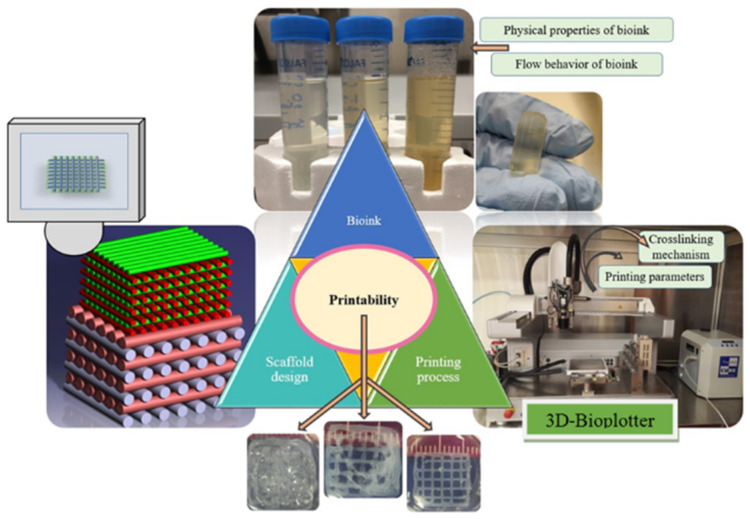
Factors affecting printability [59].

In the study of ink printability, numerous factors influence the printing outcome during the printing process. As shown in Figure 1, these factors primarily include the material composition of bioinks, the configuration of printer operational parameters, and the geometric complexity of the printed structures. These elements are intricately interconnected and collectively determine the printability of the inks. This interrelationship implies that modifying one factor can trigger a cascade of effects on the others, thereby influencing overall printability. For example, the choice of bioink materials can dictate the optimal printer parameter settings, while the structural complexity of the intended construct may necessitate specific material properties and printer configurations to ensure successful printing. Essentially, understanding and managing this complex web of interacting factors is crucial for achieving high-quality prints with consistent printability.

The rheological properties of bioinks are the core factors affecting their printability. Specifically, key rheological parameters of bioinks, such as viscosity [60,61], yield stress [62,63], storage modulus [64] and loss modulus [65], are intrinsically linked to the characteristics of the polymer materials that compose the bioink. In extrusion-based printing, inks with high viscosity are more likely to maintain their shape, but they also pose the risk of clogging the nozzle. Conversely, bioinks with a low storage modulus may compromise the structural integrity of the printed constructs due to inadequate mechanical support. The insufficient mechanical properties of such inks can lead to sagging, deformation, or even structural failure during and after printing. Existing studies have shown that optimizing the material properties of inks to regulate their rheological characteristics is crucial for achieving good printability. For example, introducing nano clay into a thermos-responsive hydrogel bioink can effectively regulate their rheological properties [66]. The modified bioinks exhibited enhanced viscoelasticity, improving the structural stacking ability and shape fidelity during printing, and further enhancing their overall printability. Another study showed that incorporating iodinated carrageenan (CG) into silk fibroin (SF) can significantly improve the viscoelastic properties of the ink [67], enabling it to exhibit excellent shape fidelity and structural stability under physiological conditions, thus achieving high printability. Therefore, fine-tuning the rheological properties of bioinks by carefully engineering their material composition is essential for achieving optimal printability. This optimization ensures that the bioink can flow smoothly through the printing nozzle while maintaining the desired shape and structural stability of the final printed product.

Among the printing parameters, key factors such as the nozzle diameter [68], printing pressure [69], printing temperature [70], and printing speed [71] play a crucial role in optimizing the printability of bioinks. The printability of a bioink based on a dual-network system of nano-hydroxyapatite/polyethylene glycol diacrylate (nHA/PEGDA) was investigated [72]. By analyzing the impact of parameters such as nozzle diameter, air pressure, and printing speed on the surface morphology of printed scaffolds, the optimal process conditions for achieving high printing precision were determined. The nozzle diameter directly impacts printing resolution: a larger diameter may reduce the risk of clogging but compromises printing accuracy. Meanwhile, a higher extrusion pressure can increase the extrusion speed, yet it generates high shear stress, which can cause irreversible damage to cells. This is especially critical when printing cell-laden bioinks.

The structure of a scaffold is closely related to its mechanical properties and biological characteristics. A regular structure can provide more uniform mechanical support, while a random structure can offer a more natural environment for cell growth [73]. By regulating the structure of the scaffold, its mechanical properties can be significantly improved, and a specific environment can be created for cell growth, differentiation, and proliferation. This is of great significance for the fields of regenerative medicine and tissue engineering. However, the design of complex scaffold structures poses higher and multi-dimensional challenges to the printability of bioinks, spanning aspects such as geometric configuration, process compatibility, and support strategies. Constructing scaffolds with complex geometries and high-precision details such as porous networks, biomimetic hierarchical structures, or finely branched vasculature requires elevated levels of printing accuracy and material stability [74,75]. In particular, when fabricating scaffolds with complex internal structures, it is often necessary to rely on additional supporting materials for the printing process [76]. The incorporation of these supporting materials can help some low printing adaptability inks manufacture complex structures, but it also introduces a large number of additional control variables, such as the relative relationship between the storage modulus (G′) of the support bath and that of the bioinks, which can significantly influence the morphological fidelity of printed filaments—a mismatch may result in filament dragging or structural rupture [77,78]. Moreover, the rheological relationship between the support bath and the bio ink can also affect the printing effect, and the complex interplay among these parameters significantly compounds the challenges associated with optimizing and regulating the printability of bioinks.

Theoretically, through the systematic modulation of the interactions among multiple parameters, high-fidelity printing of bioinks can be achieved [79]. Nevertheless, in practice, the determinants of bioink printability extend far beyond the parameters previously discussed. They also include critical aspects such as the choice of cross-linking agents, cross-linking duration, and cross-linking agent concentration [80]. These influencing factors typically exhibit nonlinear relationships with one another. This intricate web of interdependencies renders the realization of high-fidelity bioink printing an arduous and complex endeavor.

## 3. Machine Learning

Currently, numerous studies are focusing on developing prediction models using machine-learning algorithms to accurately assess the printability of bioinks. These studies gather extensive data related to bioink characteristics, printing parameters, and printability outcomes, and then train machine-learning models to predict and optimize printability under various conditions. Specifically, machine-learning algorithms can generally be categorized into supervised learning, unsupervised learning, and deep learning. Typically, different algorithm models need to be selected based on diverse application scenarios [81]. Even in the same application scenario, the performance of various algorithms varies. Therefore, it is necessary to compare the evaluation results of different algorithms to determine the most suitable algorithm model. For example, to investigate low-temperature printing technology for addressing the issue of limited preservation time of printed products, Qian Qiao [82] compared the performance of four algorithms—multiple linear regression (MLR), decision tree (DT), random forest (RF), and artificial neural network (ANN)—and determined that ANN was the optimal model. Table 3 summarizes several common algorithms used in the evaluation of the printability of inks.

As shown in Table 3, most of the models applied in 3D bioprinting are supervised learning methods. These approaches rely on the labeled data between the existing process parameters and the printing results to establish the mapping relationship between the input and the output. However, the performance of supervised learning algorithms is limited by data quality and comprehensiveness. This limitation becomes particularly pronounced in the exploration of novel materials, where algorithm performance may suffer due to insufficient training data. Looking ahead, integrating techniques such as transfer learning and semi-supervised learning holds promise for overcoming these current constraints. By capitalizing on pre-trained models and leveraging unlabeled data, researchers can potentially unlock new capabilities, thereby propelling the advancement of 3D bioprinting technology.

### 3.1. Introduction to Common Algorithms

#### 3.1.1. Bayesian Optimization

Bayesian optimization is a global optimization method for black-box models. Its core idea is to use statistical methods to transform uncertainties into probability distributions and gradually find the optimal solution of the objective function by utilizing the known information. Specifically, Bayesian optimization approximates the objective function by constructing a surrogate model (such as a Gaussian process), and selects the next sampling point based on Bayes’ theorem to maximize the objective function. The optimization process will stop when the preset optimal value is reached or the maximum number of iterations is achieved.

#### 3.1.2. Neural Network

A neural network is an algorithmic model that simulates the structure and function of biological neurons. It processes information through a large number of interconnected neuron nodes to solve complex pattern recognition and prediction problems. A typical neural network consists of an input layer, hidden layers, and an output layer. The input layer receives the raw data, the hidden layers perform non-linear transformations on the data, and the output layer generates the final prediction results. The process of data flowing from the input layer to the output layer is called forward propagation, while backpropagation calculates the error between the predicted results and the true values, and updates the network weights and biases using the chain rule, thus gradually optimizing the model’s performance.

#### 3.1.3. Random Forest

A random forest is an ensemble learning algorithm that combines multiple decision trees to solve classification and regression problems. This algorithm obtains the final prediction by aggregating the prediction results of multiple decision trees. The random forest algorithm generates multiple data subsets from the original dataset through the bootstrap sampling method, and each subset is used to independently train a decision tree, which helps to increase the diversity of the data and reduce the risk of overfitting. When constructing each node of each decision tree, a subset of features is randomly selected, and the optimal feature is chosen from the selected subset for splitting. This can increase the diversity of the model and thus improve the generalization ability of the entire forest.

#### 3.1.4. Hierarchical Machine Learning

Hierarchical machine learning uses the hierarchical concept to decompose a complex machine-learning system into multiple relatively independent layers, where each layer is responsible for specific tasks or functions, and different layers can interact through interfaces. The use of a hierarchical structure can help reduce the complexity of the system, and improve its maintainability and scalability. This machine-learning approach conducts modular design and construction of algorithms, thus making it easier to locate problems. When there is a failure in the system, targeted debugging and optimization can be carried out according to the layer where the error occurs.

#### 3.1.5. Linear Regression

Linear regression is one of the most fundamental and widely used supervised learning algorithms, primarily employed to model the linear relationship between independent and dependent variables. Its core idea is to fit a best-fit line such that the weighted linear combination of input features minimizes the error between predicted and true values. As a modeling method with strong interpretability and high computational efficiency, linear regression performs well on small-scale datasets and in scenarios where feature relationships are approximately linear. Although the model has a simple structure, linear regression is relatively sensitive to multicollinearity among features and has limited capability in handling nonlinear relationships. Therefore, in complex tasks, it is often used as a baseline model or combined with other algorithms to enhance overall performance.

#### 3.1.6. XGBoost

XGBoost is an efficient machine-learning algorithm based on the gradient boosting decision tree framework, with powerful capabilities for classification and regression modeling. This method iteratively builds multiple weak learners (typically regression trees), where each iteration’s model is used to fit the residuals of the previous prediction, thereby continuously improving the overall model’s predictive performance. Compared to traditional GBDT algorithms, XGBoost introduces regularization to control model complexity, effectively preventing overfitting, and incorporates engineering improvements such as approximate split finding, cache optimization, and parallel processing, significantly enhancing training speed and model generalization ability.

## 4. The Application of Machine Learning in the Evaluation of Printability

### 4.1. Optimization of Ink Material Performance

In the application of bioinks, different polymers have been widely studied due to their distinct properties and biological characteristics. For instance, some polymers (collagen and hydrogels) exhibit excellent biocompatibility [13,15], creating a suitable microenvironment for cell growth. Additionally, other polymers like PEG and PCL exhibit notable mechanical properties [13], which contribute to maintaining the stability of printed structures. By controlling the ratio of different polymers in the components, the rheological properties can be optimized, thereby achieving superior printing results while maintaining good biocompatibility. Optimizing the rheological properties of bioinks is a complex process. In extrusion-based printing, bioinks need to possess the characteristic of shear thinning. Shear thinning allows the ink to exhibit a relatively low viscosity under high stress, allowing it to be smoothly extruded from the nozzle. Once extruded, the ink is expected to rapidly regain its high viscosity to help maintain its shape and ensure the stability of the printed structure [94].

Traditional research on the optimization of ink rheological properties relies on a large number of experiments, which not only consumes a large amount of material resources but also requires a huge investment of time. Machine-learning technology is of great help in solving this problem. By collecting and integrating multi-dimensional data, including ink composition ratios, rheological parameters, printing conditions, and corresponding printing outcomes, researchers can utilize machine-learning algorithms to construct accurate predictive models. These models can quickly uncover the intricate relationships between the rheological properties of bioinks and their printability, allowing researchers to efficiently screen for the optimal material formulations and process parameters without the need for extensive experimentation. As a result, the performance of bioinks can be optimized and upgraded more efficiently and cost-effectively.

Jooyoung Lee et al.’s research demonstrates the potential of machine learning in the development of bioinks [95]. They took a composite bioink composed of collagen, hyaluronic acid as the research object, and used machine learning to develop a model for designing bioinks with good printability. Their study revealed the general relationship between the rheological properties of the ink and its printability, demonstrating that a high elastic modulus could improve the shape fidelity. Using a multiple regression algorithm, various formulations of natural bioinks that could provide high shape fidelity were successfully derived. This research proves the convenience of machine learning in studying the relationship between rheological properties and printability. Compared to traditional methods reliant on expert experience, machine learning significantly boosts the potential for bioink development. By predicting ink formulations, researchers can design and configure new bioinks without being limited to specific materials.

**Figure 2:**
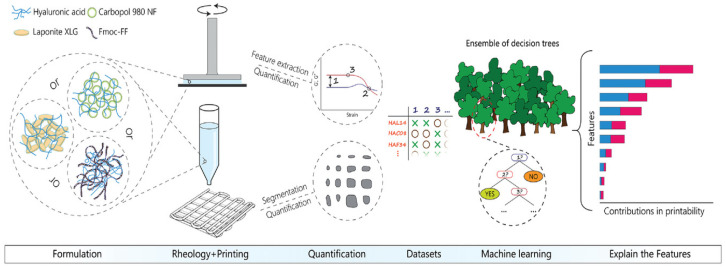
Explanation of printability using an RF algorithm [97].

Rheological modifiers, which can alter the rheological properties of inks, have been extensively utilized in bioink research and development. By incorporating these modifiers into bioinks, their rheological properties can be optimized to achieve better printability [96]. Machine learning can be used to explore the relationship between additives and printability, providing users with an interpretable guideline. One study applied machine learning to explore the relationship between the rheological index and printability [97]. In this work, a random forest (RF) model was used to analyze 180 different formulations, involving 13 key rheological measures. Given the multitude of factors influencing printability, correlation analysis was first conducted on the original dataset. Figure 2 illustrates the evaluation process of printability using the RF algorithm. Through feature selection, the data was refined to include only the features that significantly contributed to the predictive performance of the RF model. The results demonstrated that feature screening helps identify critical factors influencing model accuracy, thereby improving predictive performance. Additives were found to have varying impacts on ink printability, and machine learning-based models were effective in identifying formulations with favorable printability.

Due to their advantages in handling complex data and high-dimensional features, hierarchical machine-learning models have been widely applied in the study of bioink printability. To improve the prediction accuracy of printing resolution for printed scaffolds, a hierarchical machine-learning model based on rheological information was developed [98]. In this study, ten different bioinks were prepared using three base hydrogels and three additives. To evaluate the predictive performance of different machine-learning models, a small dataset was constructed based on these bioinks and their corresponding printing parameters. Using this dataset, three machine-learning models were trained and tested. The experimental results showed that the hierarchical machine-learning model exhibited the lowest prediction error in printing outcomes, demonstrating superior predictive performance.

These studies show that machine learning can accelerate the development of bioink formulations. By combining machine learning with experimental verification, researchers are enabled to conduct a more systematic exploration of bioink properties. This, in turn, offers more precise and functional solutions for the application of 3D bioprinting in tissue engineering and regenerative medicine. With the further optimization of machine-learning models and the increase in data volume, this approach is expected to play an even greater role in optimizing the rheological properties of bioinks and improving printing accuracy.

### 4.2. Optimization of Printing Parameters

In the research on the printability of bioinks, the parameter settings of the printer have a crucial impact on the printing quality. Factors such as printing speed, nozzle diameter, and printing temperature interact with each other and jointly affect the quality of the printed structure. In previous studies, the selection of the optimal printing parameters often relied on expert experience and continuous trial and error, which is inefficient and difficult to adapt to complex situations. With the rapid development of materials research, the properties of inks have become increasingly complex, and traditional experimental methods are unable to achieve the expected goals within a short period of time. Incorporating machine-learning algorithms into the design of printing parameters provides a new solution for the optimization of printing parameters. By summarizing and learning from a large amount of data, these algorithms enable a more sophisticated and efficient approach to achieving optimal printing conditions.

At present, data collection in the field of bioprinting heavily relies on the actual printing process. However, due to objective constraints such as lengthy printing cycles and high costs, the sample size of datasets used for training models remains significantly limited. Against this background, constructing machine-learning models suitable for limited dataset sizes is becoming a key approach. Researchers have developed a hierarchical machine-learning framework to predict high-fidelity printing parameters for alginate hydrogels [99]. This framework quantifies geometric errors between printed structures and CAD models, analyzing the coupled effects of printing speed, extrusion rate, and temperature on accuracy to dynamically assess printing performance. Notably, the algorithm shows strong robustness with limited datasets, reducing iterative experiments by 40% compared to traditional trial-and-error methods. This strategy highlights the advantages of hierarchical modeling in analyzing parameter interactions, offering new ideas to overcome small dataset limitations.

In 3D bioprinting, constructing high-fidelity structures requires precise coordination of multiple parameters, which remains a key technical challenge. Systematically analyzing parameter influence mechanisms and identifying optimal combinations are therefore critical for improving printing accuracy. A recent study [100] employed support vector machine (SVM) modeling to optimize printing parameters, investigating how factors such as nozzle specifications, temperature, and path height affect printability. As a supervised learning algorithm, SVM excels in classification, regression, and handling small-sample, high-dimensional datasets. The researchers assessed printability using a width index (ratio of measured line width to theoretical width). The resulting process diagram enabled precise selection of optimal parameter combinations, significantly enhancing the likelihood of achieving high-quality printing outcomes.

In the engineering applications of 3D bioprinting scaffolds, existing printability evaluation systems are often limited to a single performance indicator and lack a systematic consideration of multi-dimensional features such as filament morphology and interlayer pore structure. One study has proposed a comprehensive scoring method that takes filament morphology and pore quality in stacked structures as core evaluation indicators to more comprehensively reflect the printability performance of bioinks [101]. Combined with Bayesian optimization techniques, this method is used to accelerate the optimization process of extrusion printing parameters and improve the repeatability of printing results. Compared with traditional experimental design methods, this optimization strategy can significantly reduce the number of experiments and more efficiently and accurately search for optimal configurations within the preset parameter space. The results show that this method effectively optimizes printing parameters while reducing resource consumption.

Table 4 provides a summary of other applications of machine learning in optimizing printing parameters, including the machine-learning methods employed, the parameters investigated, and the indicators for evaluating printability. These research findings highlight the great potential of machine learning in printability prediction and optimization. By integrating experimental data and models, machine learning can effectively analyze the nonlinear relationships among complex parameters, significantly reducing the costs and time consumption of traditional methods. In future developments, advanced machine-learning algorithms are expected to provide more intelligent solutions for the screening of printable inks and the optimization of printing parameters.

### 4.3. Applications in Predicting Cell Viability

The ultimate goal of bioprinting is to fabricate 3D scaffolds with biological functions. In practical applications, cell viability is one of the key indicators for evaluating the biocompatibility of the printed structures. Cell viability is affected by various factors, which are similar to those influencing printability, including material properties, the setting of printing parameters, and the geometric shape of the printed structure.

**Figure 3:**
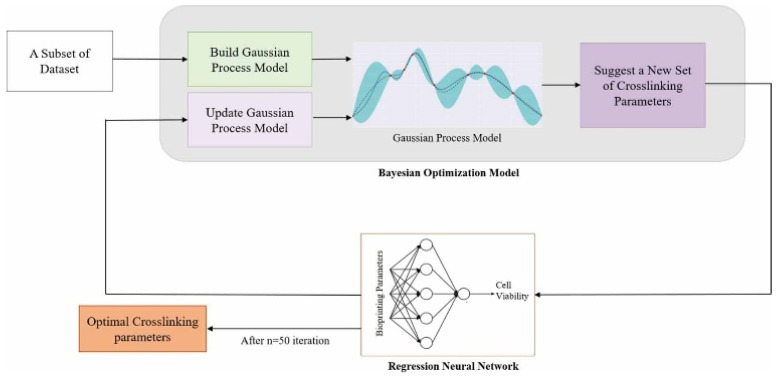
Algorithm of Bayesian optimization model based on regression neural network [107].

Figure 3 illustrates a predictive model that integrates Bayesian optimization with a neural network [107], designed to forecast cell viability based on various printing parameters. In this study, gelatin and sodium alginate were used as bioinks to construct the dataset required for model training and validation. The study systematically analyzed the effects of factors such as material concentration, printing speed, nozzle size, and cartridge temperature on the viability of multiple cell lines. The results demonstrated that the proposed optimization model exhibits significant advantages in parameter tuning, enabling high-precision identification of optimal printing conditions and effectively enhancing cell viability.

In the process of extrusion-based 3D bioprinting, shear stress is one of the key factors affecting cell viability. Therefore, it is necessary to systematically study the relationship between shear stress and cell damage and determine the appropriate shear stress threshold to maximize cell integrity while ensuring printing accuracy. However, current studies are mostly focused on a limited number of cell lines, lacking broad applicability and generalizability. To address this issue, a quantitative analysis framework combining numerical simulation and machine learning has been developed to evaluate the effect of shear stress on the survival rate of different types of cells during the extrusion process [108]. This framework integrates support vector regression (SVR) with computational fluid dynamics simulations and, based on experimental data from various cell types, trains a multilayer perceptron (MLP) regression model to predict cell survival rates according to the magnitude and duration of shear stress experienced by the cells. This method demonstrates good generalizability and can be applied to different types of bioinks and cell types, providing effective theoretical support for parameter optimization and cell viability prediction in bioprinting processes, thereby promoting the development of bioprinting technology.

These studies indicate that machine learning not only has great application prospects in printability prediction but also possesses enormous application potential in other aspects of bioprinting. In future research and development, the scalability of machine learning should be fully utilized, and it should be combined with other research methods such as finite element simulation and numerical simulation to fully unleash the potential of machine learning and open up a broader path for the development of 3D bioprinting technology.

## 5. Challenges and Opportunities

With the deep integration of machine learning and 3D bioprinting technology, significant progress has been made in several key areas, including printability prediction, optimization of printing parameters, cell viability prediction, and scaffold performance assessment. However, the application of machine learning in the evaluation of the printability of polymers remains fraught with challenges, which are mainly concentrated in the following aspects.

a. At the data level

Firstly, the performance of machine-learning models highly depends on the quality of the dataset. In current research, the machine-learning algorithms used by experimenters mainly focus on supervised learning. These models require users to train the model with labeled data. However, the scale and diversity of the dataset make labeling extremely difficult. Manual labeling is time-consuming and labor-intensive, and automatic labeling cannot guarantee the accuracy of the labels. These issues collectively increase the difficulty of obtaining high-quality data.

Secondly, there is still no unified standard for data collection in 3D bioprinting. With the continuous advancement of biological materials and the rapid development of printing technology, research interests have diversified significantly. In the absence of a unified framework, experimenters lack clear directives on data collection. This disparity results in substantial variations in datasets across different studies, even when investigating the same materials. Such inconsistencies render it difficult, if not impossible, to utilize publicly available datasets from other research groups for model training, thereby limiting the reproducibility and generalizability of research findings.

Feature selection within datasets is another critical challenge. As noted, the number of factors influencing printability is vast, and each feature exerts a distinct influence on the model. The dimensionality of features can significantly impact algorithm performance, necessitating the strategic selection and elimination of less relevant features to optimize model efficiency. Striking an appropriate balance between the number of features and model performance represents a significant hurdle in data processing, requiring careful consideration and advanced analytical techniques.

b. At the model level

Although the types of machine-learning models used in the field of 3D bioprinting are continuously increasing, most of the research still focuses on the training and testing of a specific material. This narrow-focus approach undermines the models’ generalization ability. Consequently, when researchers embark on studying new materials, they often find themselves compelled to redesign the model and optimize its structural parameters from scratch. This iterative process not only inflates research costs but also demands substantial time investments, impeding the pace of technological advancement.

Moreover, the black-box nature of machine learning, particularly pronounced in deep-learning models, leads to a lack of interpretability. These models typically lack transparent and intuitive explanations, leaving users unable to comprehensively grasp the causal links between feature inputs and output results. This opacity severely restricts researchers’ ability to fully understand and validate the prediction mechanisms, thereby hampering the refinement and reliable application of these models in 3D bioprinting research.

c. At the application level

Machine learning holds great promise in predicting printing quality and optimizing process parameters, thereby substantially enhancing research efficiency. Nevertheless, in applications, model deployment remains a formidable task, with limited integration between machine-learning models and existing printing systems. Moreover, the dearth of mature application platforms exacerbates these challenges.

For researchers without a strong programming background, leveraging algorithmic programs poses an additional hurdle. Developing a user-friendly visual interface to facilitate their experimentation is imperative, yet this requirement places stringent demands on human-computer interaction design. Such challenges underscore the need for more accessible, integrated, and intuitive solutions to fully realize the potential of machine learning in 3D bioprinting applications.

## 6. Future Outlook

With the continuous development of science and technology, the application of machine-learning in 3D bioprinting will exhibit a new development trend. On the one hand, deep learning will persist as a cornerstone, undergoing continuous refinement and innovation. Its potential shines brightest in image-related applications, such as defect detection. Deep-learning models enable highly accurate and automated quality inspection, thereby revolutionizing inspection efficiency. Concurrently, the rapid progress of transfer learning will markedly boost model generalization. These enhanced models can effectively discern long-range correlations among bioink properties, printing parameters, and printability, offering more precise guidance for optimizing the printing process.

On the other hand, the integration of multimodal data is emerging as a key research focus. 3D bioprinting encompasses a diverse array of data types, such as the physicochemical characteristics of bioinks, imaging data captured during printing, and cell viability metrics. The fusion of these multimodal datasets enriches the information available to machine-learning algorithms, thereby bolstering model performance and interpretability. Moreover, the application of generative adversarial networks (GANs) has revolutionized 3D bioprinting design. By incorporating data on printing parameters and CAD models, GANs can produce highly realistic depictions of printed constructs and visually compare the outcomes before and after optimization, significantly diminishing the necessity for iterative experimentation.

## 7. Conclusions

Recent studies have shown that the combination of machine learning and 3D bioprinting has important application value in printability evaluation and optimization. By constructing predictive models, analyzing influencing factors, and optimizing printing strategies, machine learning provides powerful support for improving the quality and efficiency of 3D bioprinting. Some successful cases have been achieved in relevant research, and applications have been made in fields such as tissue engineering and regenerative medicine. However, there are still challenges such as the quality and quantity of data, and the complexity and interpretability of models. In the future, with the development of technology, the application of machine learning in the evaluation of the printability of 3D bioprinting will continue to be improved, and it is expected to be extended to broaden fields, bringing more innovations and breakthroughs to the fields of biomanufacturing and medicine. In order to fully unleash the potential of machine learning, it is necessary to further strengthen interdisciplinary research and promote the widespread application and development of 3D bioprinting technology.

## Figures and Tables

**Table 1:** Common bioink materials.

Biomaterials	Representative Materials	Advantages/Disadvantages	Application	Ref.
Natural Polymers	Collagen, Chitosan, Alginate, Silk fibroin, Gelatin, Hyaluronic Acid (HA), GelHA, Agarose	Advantages: Bioactive Biocompatible Disadvantages: Low mechanical strength Limited processability	Drug delivery Cell culture medium Biological scaffolds Biosensors Wound dressing	[10,11,12,13]
Synthetic Polymers	Polyethylene Glycol (PEG), Polycaprolactone (PCL), Polylactic Acid (PLA), Polyvinyl Alcohol (PVA), Polyacrylamide (PAM), Polydopamine (PDA)	Advantages: Tunable mechanical strength Process versatility Disadvantages: Limited cell interactions Poor cell adhesion	Soft tissue repair Biosensors Biological scaffolds Medical textiles Drug delivery Absorbable sutures	[13,14,15]
Metallic Materials	Stainless Steel, Titanium and Titanium Alloys, Magnesium Alloys, Cobalt-Chromium Alloys, Tantalum, Nickel Titanium Alloys	Advantages: Excellent mechanical strength Customizable mechanical properties Disadvantages: Expensive and Hard to process	Bone screws Vascular stents Dental implants Neurostimulators Medical tubing Artificial hip	[16,17,18,19]
Ceramic Materials	Alumina, Bioactive Glass, β-Tricalcium Phosphate, Hydroxyapatite (HA), Calcium Silicate	Advantages: High biocompatibility Excellent bioactivity Disadvantages: Low fracture toughness Difficult to process	Filling material Implant coatings Biosensors Ceramic medical membranes	[20,21,22,23]
Composite Materials	PLA/HA, Gelatin/HA, Collagen/PEG, Chitosan/Hydroxyapatite Gelatin/PCL, PCL/Bioactive Glass	Advantages: Combined properties of components High biocompatibility Synergistic effects Disadvantages: Complex processing Higher cost	Orthopedic implants Soft tissue repair Drug delivery Biological scaffolds Wound dressing	[24,25,26,27]

**Table 2:** Common printability evaluation methods.

Evaluation Method	Evaluation Content	Definition	Ref.
Extrudability	Forming continuous filaments	Line continuity during extrusion	[47,48,49]
Angular fidelity factor	AF = $\frac{Filament\;thickness}{tip\;thickness}$	Assessing complex angle variations	[50]
Width index	WI = $\frac{average\;width}{theoretical\;width}$	Filament change relative to nozzle diameter	[51,52]
Printability index	Pr = $\frac{{perimeter}^{2}}{16\times area}$	Square pore matching in scaffold design	[53,54,55]
Integrity factor	I = $\;\frac{Scaffold\;thickness}{Control\;thickness}$	Thickness comparison	[56,57]
Irregularity	Irregularity = $\frac{\left|{experiment}_{X,Y,Z}\right|}{{design\;length}_{X,Y,Z}}$	3D structural integrity index	[58,59]

**Table 3:** Comparison of the advantages and disadvantages of common machine-learning algorithms in existing research.

Algorithm	Type	Advantages	Limitations	Ref.
Linear Regression	Supervised Learning	Simple model Fast training	Struggles with nonlinear relationships Sensitive to outliers	[83]
Random Forest	Supervised Learning	Feature interpretability Strong resistance to overfitting	Complex hyperparameter tuning Demonstrates overfitting tendencies for small-scale data	[84,85]
XGBoost	Supervised Learning	High accuracy Fast training	Sensitive to outliers Complex hyperparameter tuning	[86]
Bayesian Algorithm	Supervised Learning	Friendly for small samples Highly interpretable	High computational complexity Difficulties in sampling from multimodal distributions	[87,88,89]
Neural Network	Deep Learning	Strong expressive power Strong nonlinear modeling capability	High data dependence Limited model transparency	[90,91,92]
Hierarchical Machine Learning	Supervised Learning	Hierarchical data processing capability Phase-wise prediction	Computationally intensive training Precise hierarchical configuration	[93]

**Table 4:** Applications of algorithmic models, parameter optimization, and evaluation indicators of machine learning in the 3D printing of various biological materials.

Main Materials	Parameters	Algorithm Model	Print Assessment	Ref.
Alginate and CMC	Printing speed Printing pressure Printing distance Nozzle diameter Viscosity	Regression model	Filament width and porosity	[83]
Silicone elastomers	Nozzle diameter Extruded velocity Needle retraction distance	Hierarchical machine learning	Printing score based on layer fusion, stringing and filling volume components	[93]
Gelatin	Printing pressure Nozzle speed Printing distance	Convolutional Neural Network (CNN)	Line width, droplet line	[102]
Alginate, CMC and TO-NFC	Material concentration Shear rate	Multiple linear regression	Filament width and cell viability	[103]
Chitosan, agarose and gelatin	Printing temperature Printing speed Printing pressure Material concentration	Bayesian optimization	Subjective evaluation of printed layer and pore structure	[104]
Gelatin and sodium alginate	Material concentration Printing speed Printing pressure	Multilayer perceptron (MLP)	Evaluation of pore connectivity and shape fidelity using diffusion ratio	[105]
Alginate	Material concentration Pressure	Convolutional Neural Network (CNN)	Judgement based on filament thickness uniformity and hydrogel distribution uniformity	[106]

## Data Availability

Data are contained within the article.

## References

[1] Zhu Z., Ng D.W.H., Park H.S., McAlpine M.C. (2020). 3D-printed multifunctional materials enabled by artificial-intelligence-assisted fabrication technologies. Nat. Rev. Mater..

[2] Zhang Z., Zhou X., Fang Y., Xiong Z., Zhang T. (2025). AI-driven 3D bioprinting for regenerative medicine: From bench to bedside. Bioact. Mater..

[3] Sun J., Yao K., An J., Jing L., Huang K., Huang D. (2024). Machine learning and 3D bioprinting. Int. J. Bioprint..

[4] Ji S., Guvendiren M. (2021). Complex 3D bioprinting methods. APL Bioeng..

[5] Ramesh S., Deep A., Tamayol A., Kamaraj A., Mahajan C., Madihally S. (2024). Advancing 3D bioprinting through machine learning and artificial intelligence. Bioprinting.

[6] Gu Z., Fu J., Lin H., He Y. (2020). Development of 3D bioprinting: From printing methods to biomedical applications. Asian J. Pharm. Sci..

[7] Abu Owida H. (2022). Developments and Clinical Applications of Biomimetic Tissue Regeneration using 3D Bioprinting Technique. Appl. Bion. Biomech..

[8] Wang H., Bi S., Shi B., Ma J., Lv X., Qiu J., Wei Y. (2023). Recent Advances in Engineering Bioinks for 3D Bioprinting. Adv. Eng. Mater..

[9] Hospodiuk M., Dey M., Sosnoski D., Ozbolat I.T. (2017). The bioink: A comprehensive review on bioprintable materials. Biotechnol. Adv..

[10] Li H., Yin Y., Xiang Y., Liu H., Guo R. (2020). A novel 3D printing PCL/GelMA scaffold containing USPIO for MRI-guided bile duct repair. Biomed. Mater..

[11] Mendoza-Cerezo L., Rodríguez-Rego J.M., Macías-García A., Callejas-Marín A., Sánchez-Guardado L., Marcos-Romero A.C. (2024). Three-Dimensional Bioprinting of GelMA Hydrogels with Culture Medium: Balancing Printability, Rheology and Cell Viability for Tissue Regeneration. Polymers.

[12] Wang W., Zhu Y., Liu Y., Chen B., Li M., Yuan C., Wang P. (2024). 3D bioprinting of DPSCs with GelMA hydrogel of various concentrations for bone regeneration. Tissue Cell.

[13] Moon S.H., Choi H.N., Yang Y.J. (2022). Natural/Synthetic Polymer Materials for Bioink Development. Biotechnol. Bioprocess Eng..

[14] Negut I., Dorcioman G., Grumezescu V. (2020). Scaffolds for Wound Healing Applications. Polymers.

[15] Satchanska G., Davidova S., Petrov P.D. (2024). Natural and Synthetic Polymers for Biomedical and Environmental Applications. Polymers.

[16] Bandyopadhyay A., Mitra I., Goodman S.B., Kumar M., Bose S. (2023). Improving biocompatibility for next generation of metallic implants. Prog. Mater. Sci..

[17] Rotella G., Morano C., Saffioti M.R., Umbrello D. (2024). Surface functionalization of titanium screws for orthopaedic implant applications. CIRP Ann..

[18] Al-Shalawi F.D., Mohamed Ariff A.H., Jung D.-W., Mohd Ariffin M.K.A., Seng Kim C.L., Brabazon D., Al-Osaimi M.O. (2023). Biomaterials as Implants in the Orthopedic Field for Regenerative Medicine: Metal versus Synthetic Polymers. Polymers.

[19] Kim S.H., Ki M.-R., Han Y., Pack S.P. (2024). Biomineral-Based Composite Materials in Regenerative Medicine. Int. J. Mol. Sci..

[20] Ferrández-Montero A., Ortega-Columbrans P., Eguiluz A., Sanchez-Herencia A.J., Detsch R., Boccaccini A.R., Ferrari B. (2024). Biocompatible colloidal feedstock for material extrusion processing of bioceramic-based scaffolds. Polym. Compos..

[21] Rahimnejad M., Rezvaninejad R., Rezvaninejad R., França R. (2021). Biomaterials in bone and mineralized tissue engineering using 3D printing and bioprinting technologies. Biomed. Phys. Eng. Express.

[22] Ana I.D., Satria G.A.P., Dewi A.H., Ardhani R., Chun H.J., Park K., Kim C.-H., Khang G. (2018). Bioceramics for Clinical Application in Regenerative Dentistry. Novel Biomaterials for Regenerative Medicine.

[23] Joddar B., Ito Y. (2011). Biological modifications of materials surfaces with proteins for regenerative medicine. J. Mater. Chem..

[24] Kuperkar K., Atanase L., Bahadur A., Crivei I., Bahadur P. (2024). Degradable Polymeric Bio(nano)materials and Their Biomedical Applications: A Comprehensive Overview and Recent Updates. Polymers.

[25] Cui X., Li J., Hartanto Y., Durham M., Tang J., Zhang H., Hooper G., Lim K., Woodfield T. (2020). Advances in Extrusion 3D Bioprinting: A Focus on Multicomponent Hydrogel-Based Bioinks. Adv. Healthc. Mater..

[26] Zhang S., Chen X., Shan M., Hao Z., Zhang X., Meng L., Zhai Z., Zhang L., Liu X., Wang X. (2023). Convergence of 3D Bioprinting and Nanotechnology in Tissue Engineering Scaffolds. Biomimetics.

[27] Loukelis K., Helal Z.A., Mikos A.G., Chatzinikolaidou M. (2023). Nanocomposite Bioprinting for Tissue Engineering Applications. Gels.

[28] Gómez-Blanco J.C., Pagador J.B., Galván-Chacón V.P., Sánchez-Peralta L.F., Matamoros M., Marcos A., Sánchez-Margallo F.M. (2024). Computational simulation-based comparative analysis of standard 3D printing and conical nozzles for pneumatic and piston-driven bioprinting. Int. J. Bioprint..

[29] Ning L., Chen X. (2017). A brief review of extrusion-based tissue scaffold bio-printing. Biotechnol. J..

[30] Ozbolat I.T., Hospodiuk M. (2016). Current advances and future perspectives in extrusion-based bioprinting. Biomaterials.

[31] Yang Y., Wang X., Lin X., Xie L., Ivone R., Shen J., Yang G. (2020). A tunable extruded 3D printing platform using thermo-sensitive pastes. Int. J. Pharm..

[32] Koch F., Thaden O., Conrad S., Tröndle K., Finkenzeller G., Zengerle R., Kartmann S., Zimmermann S., Koltay P. (2022). Mechanical properties of polycaprolactone (PCL) scaffolds for hybrid 3D-bioprinting with alginate-gelatin hydrogel. J. Mech. Behav. Biomed. Mater..

[33] Ji H., Zhang X., Huang X., Zheng L., Ye X., Li Y. (2019). Effect of extrusion on viscoelastic slurry 3D print quality: Numerical analysis and experiment validation. SN Appl. Sci..

[34] Yogeshwaran S., Goodarzi Hosseinabadi H., Gendy D.E., Miri A.K. (2024). Design considerations and biomaterials selection in embedded extrusion 3D bioprinting. Biomater. Sci..

[35] Schwab A., Levato R., D’Este M., Piluso S., Eglin D., Malda J. (2020). Printability and Shape Fidelity of Bioinks in 3D Bioprinting. Chem. Rev..

[36] Curti F., Drăgușin D.-M., Serafim A., Iovu H., Stancu I.-C. (2021). Development of thick paste-like inks based on superconcentrated gelatin/alginate for 3D printing of scaffolds with shape fidelity and stability. Mater. Sci. Eng. C.

[37] Khattati M., Abarghooei E., Hemasian Etefagh A., Khajehzadeh M., Razfar M.R. (2025). Experimental investigation of pre-crosslinking methods and employing nano-hydroxyapatite powder on alginate/carboxymethyl cellulose hydrogel printability via 3D bioprinting. Rapid Prototyp. J..

[38] Freeman S., Calabro S., Williams R., Jin S., Ye K. (2022). Bioink Formulation and Machine Learning-Empowered Bioprinting Optimization. Front. Bioeng. Biotechnol..

[39] Yu C., Jiang J. (2020). A Perspective on Using Machine Learning in 3D Bioprinting. Int. J. Bioprinting.

[40] Wu D., Wei Y., Terpenny J. (2018). Surface Roughness Prediction in Additive Manufacturing Using Machine Learning. Volume 3: Manufacturing Equipment and Systems, Proceedings of the ASME 2018 13th International Manufacturing Science and Engineering Conference, College Station, TX, USA, 18–22 June 2018.

[41] Ege D., Boccaccini A.R. (2024). Investigating the Effect of Processing and Material Parameters of Alginate Dialdehyde-Gelatin (ADA-GEL)-Based Hydrogels on Stiffness by XGB Machine Learning Model. Bioengineering.

[42] Shi J., Song J., Song B., Lu W.F. (2019). Multi-Objective Optimization Design through Machine Learning for Drop-on-Demand Bioprinting. Engineering.

[43] Entekhabi E., Haghbin Nazarpak M., Sedighi M., Kazemzadeh A. (2020). Predicting degradation rate of genipin cross-linked gelatin scaffolds with machine learning. Mater. Sci. Eng..

[44] Shirmohammadi M., Goushchi S.J., Keshtiban P.M. (2021). Optimization of 3D printing process parameters to minimize surface roughness with hybrid artificial neural network model and particle swarm algorithm. Prog. Addit. Manuf..

[45] Saad M.S., Nor A.M., Baharudin M.E., Zakaria M.Z., Aiman A. (2019). Optimization of surface roughness in FDM 3D printer using response surface methodology, particle swarm optimization, and symbiotic organism search algorithms. Int. J. Adv. Manuf. Technol..

[46] Lee S.C., Gillispie G., Prim P., Lee S.J. (2020). Physical and Chemical Factors Influencing the Printability of Hydrogel-based Extrusion Bioinks. Chem. Rev..

[47] Fu Z., Naghieh S., Xu C., Wang C., Sun W., Chen X. (2021). Printability in extrusion bioprinting. Biofabrication.

[48] Wilson S.A., Cross L.M., Peak C.W., Gaharwar A.K. (2017). Shear-Thinning and Thermo-Reversible Nanoengineered Inks for 3D Bioprinting. ACS Appl. Mater. Interfaces.

[49] Roehm K.D., Madihally S.V. (2017). Bioprinted chitosan-gelatin thermosensitive hydrogels using an inexpensive 3D printer. Biofabrication.

[50] Perin F., Spessot E., Famà A., Bucciarelli A., Callone E., Mota C., Motta A., Maniglio D. (2023). Modeling a Dynamic Printability Window on Polysaccharide Blend Inks for Extrusion Bioprinting. ACS Biomater. Sci. Eng..

[51] Kang K.H., Hockaday L.A., Butcher J.T. (2013). Quantitative optimization of solid freeform deposition of aqueous hydrogels. Biofabrication.

[52] Li Q., Zhang B., Xue Q., Zhao C., Luo Y., Zhou H., Ma L., Yang H., Bai D. (2021). A Systematic Thermal Analysis for Accurately Predicting the Extrusion Printability of Alginate–Gelatin-Based Hydrogel Bioinks. Int. J. Bioprint..

[53] Coşkun S., Akbulut S.O., Sarıkaya B., Çakmak S., Gümüşderelioğlu M. (2022). Formulation of chitosan and chitosan-nanoHAp bioinks and investigation of printability with optimized bioprinting parameters. Int. J. Biol. Macromol..

[54] Sarker M., Izadifar M., Schreyer D., Chen X. (2018). Influence of ionic crosslinkers (Ca^2+^/Ba^2+^/Zn^2+^) on the mechanical and biological properties of 3D Bioplotted Hydrogel Scaffolds. J. Biomater. Sci. Polym. Ed..

[55] Alarçin E., Tutar R., Titi K., Kocaaga B., Guner F.S., Bal-Öztürk A. (2023). Optimization of methacrylated gelatin/layered double hydroxides nanocomposite cell-laden hydrogel bioinks with high printability for 3D extrusion bioprinting. J. Biomed. Mater. Res..

[56] Malekpour A., Chen X. (2022). Printability and Cell Viability in Extrusion-Based Bioprinting from Experimental, Computational, and Machine Learning Views. J. Funct. Biomater..

[57] Soltan N., Ning L., Mohabatpour F., Papagerakis P., Chen X. (2019). Printability and Cell Viability in Bioprinting Alginate Dialdehyde-Gelatin Scaffolds. ACS Biomater. Sci. Eng..

[58] Naghieh S., Chen X. (2021). Printability–A key issue in extrusion-based bioprinting. J. Pharm. Anal..

[59] Naghieh S., Sarker M., Sharma N.K., Barhoumi Z., Chen X. (2019). Printability of 3D Printed Hydrogel Scaffolds: Influence of Hydrogel Composition and Printing Parameters. Appl. Sci..

[60] Limon S., Sarah R., Habib M.A. (2024). Viscosity Inference of Hybrid Bioink Using Decision Tree-Based Machine Learning Method. Volume 1: Additive Manufacturing; Advanced Materials Manufacturing; Biomanufacturing; Life Cycle Engineering, Proceedings of the ASME 2024 19th International Manufacturing Science and Engineering Conference, Knoxville, TN, USA, 17–21 January 2024.

[61] Zhu F., Cheng L., Yin J., Wu Z.L., Qian J., Fu J., Zheng Q. (2016). 3D Printing of Ultratough Polyion Complex Hydrogels. ACS Appl. Mater. Interfaces.

[62] Kiyotake E.A., Douglas A.W., Thomas E.E., Nimmo S.L., Detamore M.S. (2019). Development and quantitative characterization of the precursor rheology of hyaluronic acid hydrogels for bioprinting. Acta Biomater..

[63] Mouser V.H.M., Melchels F.P.W., Visser J., Dhert W.J.A., Gawlitta D., Malda J. (2016). Yield stress determines bioprintability of hydrogels based on gelatin-methacryloyl and gellan gum for cartilage bioprinting. Biofabrication.

[64] López-Marcial G.R., Zeng A.Y., Osuna C., Dennis J., García J.M., O’Connell G.D. (2018). Agarose-Based Hydrogels as Suitable Bioprinting Materials for Tissue Engineering. ACS Biomater. Sci. Eng..

[65] Gao T., Gillispie G.J., Copus J.S., Pr A.K., Seol Y.-J., Atala A., Yoo J.J., Lee S.J. (2018). Optimization of gelatin–alginate composite bioink printability using rheological parameters: A systematic approach. Biofabrication.

[66] Hu C., Hahn L., Yang M., Altmann A., Stahlhut P., Groll J., Luxenhofer R. (2021). Improving printability of a thermoresponsive hydrogel biomaterial ink by nanoclay addition. J. Mater. Sci..

[67] Moon S.H., Park T.Y., Cha H.J., Yang Y.J. (2024). Photo-/thermo-responsive bioink for improved printability in extrusion-based bioprinting. Mater. Today Bio.

[68] Chirianni F., Vairo G., Marino M. (2024). Development of process design tools for extrusion-based bioprinting: From numerical simulations to nomograms through reduced-order modeling. Comput. Methods Appl. Mech. Eng..

[69] Munoz-Perez E., Perez-Valle A., Igartua M., Santos-Vizcaino E., Hernandez R.M. (2023). High resolution and fidelity 3D printing of Laponite and alginate ink hydrogels for tunable biomedical applications. Biomater. Adv..

[70] Ouyang L. (2016). Effect of bioink properties on printability and cell viability for 3D bioplotting of embryonic stem cells. Biofabrication.

[71] Webb B., Doyle B.J. (2017). Parameter optimization for 3D bioprinting of hydrogels. Bioprinting.

[72] Li Y., Ma J., Wang J., Kong Y., Wang F., Zhang P., Fan Y. (2025). Optimal parameter setting and evaluation for ultraviolet-assisted direct ink writing bioprinting of nHA/PEGDA scaffold. Biomed. Mater..

[73] Bin Rashid A., Uddin A.S.M.N., Azrin F.A., Saad K.S.K., Hoque M.E. (2023). 3D bioprinting in the era of 4th industrial revolution–insights, advanced applications, and future prospects. Rapid Prototyp. J..

[74] Rahmani Dabbagh S., Ozcan O., Tasoglu S. (2022). Machine learning-enabled optimization of extrusion-based 3D printing. Methods.

[75] Gillispie G.J. (2023). The correlation between rheological properties and extrusion-based printability in bioink artifact quantification. Mater. Des..

[76] Chaurasia P., Singh R., Mahto S.K. (2024). FRESH-based 3D bioprinting of complex biological geometries using chitosan bioink. Biofabrication.

[77] Brunel L.G., Hull S.M., Heilshorn S.C. (2022). Engineered assistive materials for 3D bioprinting: Support baths and sacrificial inks. Biofabrication.

[78] Grosskopf A.K., Truby R.L., Kim H., Perazzo A., Lewis J.A., Stone H.A. (2018). Viscoplastic Matrix Materials for Embedded 3D Printing. ACS Appl. Mater. Interfaces.

[79] Dorati R., Chiesa E., Riva F., Modena T., Marconi S., Auricchio F., Genta I., Conti B. (2022). Design and optimization of 3D-bioprinted scaffold framework based on a new natural polymeric bioink. J. Pharm. Pharmacol..

[80] Giuseppe M.D., Law N., Webb B., Macrae R.A., Liew L.J., Sercombe T.B., Dilley R.J., Doyle B.J. (2018). Mechanical behaviour of alginate-gelatin hydrogels for 3D bioprinting. J. Mech. Behav. Biomed. Mater..

[81] Krishna D.V., Sankar M.R. (2023). Machine learning-assisted extrusion-based 3D bioprinting for tissue regeneration applications. Ann. 3D Print. Med..

[82] Qiao Q. (2023). The use of machine learning to predict the effects of cryoprotective agents on the GelMA-based bioinks used in extrusion cryobioprinting. Bio-Des. Manuf..

[83] Limon S.M., Quigley C., Sarah R., Habib A. (2024). Advancing scaffold porosity through a machine learning framework in extrusion based 3D bioprinting. Front. Mater..

[84] Huang X., Ng W.L., Yeong W.Y. (2024). Predicting the number of printed cells during inkjet-based bioprinting process based on droplet velocity profile using machine learning approaches. J. Intell. Manuf..

[85] Chen H., Liu Y., Balabani S., Hirayama R., Huang J. (2023). Machine Learning in Predicting Printable Biomaterial Formulations for Direct Ink Writing. Research.

[86] Ege D., Sertturk S., Acarkan B., Ademoglu A. (2023). Machine learning models to predict the relationship between printing parameters and tensile strength of 3D Poly (lactic acid) scaffolds for tissue engineering applications. Biomed. Phys. Eng. Express.

[87] Etefagh A.H., Razfar M.R. (2023). Bayesian optimization of 3D bioprinted polycaprolactone/magnesium oxide nanocomposite scaffold using a machine learning technique. Proc. Inst. Mech. Eng. B J. Eng. Manuf..

[88] Chen S.-L., Senadeera M., Ruberu K., Chung J., Rana S., Venkatesh S., Chen C.-Y., Chen G.-Y., Wallace G. (2024). Machine learning-generated compression modulus database for 3D printing of gelatin methacryloyl. Int. J. Bioprint..

[89] Xu Y., Sarah R., Habib A., Liu Y., Khoda B. (2024). Constraint based Bayesian optimization of bioink precursor: A machine learning framework. Biofabrication.

[90] Li Z. (2021). Predicting bone regeneration from machine learning. Nat. Comput. Sci..

[91] Khalvandi A., Saber-Samandari S., Aghdam M.M. (2022). Application of artificial neural networks to predict Young’s moduli of cartilage scaffolds: An in-vitro and micromechanical study. Biomater. Adv..

[92] Jin Z., Zhang Z., Shao X., Gu G.X. (2023). Monitoring Anomalies in 3D Bioprinting with Deep Neural Networks. ACS Biomater. Sci. Eng..

[93] Menon A., Póczos B., Feinberg A.W., Washburn N.R. (2019). Optimization of Silicone 3D Printing with Hierarchical Machine Learning. 3D Print. Addit. Manuf..

[94] Sedigh A., Ghelich P., Quint J., Mollocana Lara E.C., Samandari M., Tamayol A., Tomlinson R.E. (2023). Approximating scaffold printability utilizing computational methods. Biofabrication.

[95] Lee J., Oh S.J., An S.H., Kim W.-D., Kim S.-H. (2020). Machine learning-based design strategy for 3D printable bioink: Elastic modulus and yield stress determine printability. Biofabrication.

[96] Decante G., Costa J.B., Silva-Correia J., Collins M.N., Reis R.L., Oliveira J.M. (2021). Engineering bioinks for 3D bioprinting. Biofabrication.

[97] Nadernezhad A., Groll J. (2022). Machine Learning Reveals a General Understanding of Printability in Formulations Based on Rheology Additives. Adv. Sci..

[98] Oh D., Shirzad M., Chang Kim M., Chung E.-J., Nam S.Y. (2023). Rheology-informed hierarchical machine learning model for the prediction of printing resolution in extrusion-based bioprinting. Int. J. Bioprint..

[99] Bone J.M., Childs C.M., Menon A., Póczos B., Feinberg A.W., LeDuc P.R., Washburn N.R. (2020). Hierarchical Machine Learning for High-Fidelity 3D Printed Biopolymers. ACS Biomater. Sci. Eng..

[100] Fu Z., Angeline V., Sun W. (2021). Evaluation of Printing Parameters on 3D Extrusion Printing of Pluronic Hydrogels and Machine Learning Guided Parameter Recommendation. Int. J. Bioprint..

[101] Ruberu K., Senadeera M., Rana S., Gupta S., Chung J., Yue Z., Venkatesh S., Wallace G. (2021). Coupling machine learning with 3D bioprinting to fast track optimisation of extrusion printing. Appl. Mater. Today.

[102] Chen B., Dong J., Ruelas M., Ye X., He J., Yao R., Fu Y., Liu Y., Hu J., Wu T. (2022). Artificial Intelligence-Assisted High-Throughput Screening of Printing Conditions of Hydrogel Architectures for Accelerated Diabetic Wound Healing. Adv. Funct. Mater..

[103] Habib A., Sarah R., Tuladhar S., Khoda B., Limon S.M. (2024). Modulating rheological characteristics of bio-ink with component weight and shear rate for enhanced bioprinted scaffold fidelity. Bioprinting.

[104] Hashemi A., Ezati M., Zumberg I., Vicar T., Chmelikova L., Cmiel V., Provaznik V. (2024). Characterization and optimization of a biomaterial ink aided by machine learning-assisted parameter suggestion. Mater. Today Commun..

[105] Dai C., Sun Y., Zhang H., Yuan Z., Zhang B., Xie Z., Li P., Liu H. (2025). New Strategies for High Efficiency and Precision Bioprinting by DOE Technology and Machine Learning. Adv. Mater. Technol..

[106] Allencherry J., Pradeep N., Shrivastava R., Joy L., Imbriacco F., Özel T. (2022). Investigation of Hydrogel and Gelatin Bath Formulations for Extrusion-Based 3D Bioprinting using Deep Learning. Procedia CIRP.

[107] Mohammadrezaei D., Podina L., Silva J.D., Kohandel M. (2024). Cell viability prediction and optimization in extrusion-based bioprinting via neural network-based Bayesian optimization models. Biofabrication.

[108] Zhang C., Elvitigala K.C.M.L., Mubarok W., Okano Y., Sakai S. (2024). Machine learning-based prediction and optimisation framework for as-extruded cell viability in extrusion-based 3D bioprinting. Virtual Phys. Prototyp..

